# Using a mixed method to develop consensus-based aims, contents, intended learning outcomes, teaching, and evaluation methods for a course on epilepsy for postgraduate or continuing education in community health nursing programs

**DOI:** 10.1186/s12909-021-03001-2

**Published:** 2021-11-12

**Authors:** Ramzi Shawahna

**Affiliations:** 1 Department of Physiology, Pharmacology and Toxicology, Faculty of Medicine and Health Sciences, An-Najah National University, New Campus, Building: 19, Office: 1340, P.O. Box 7, Nablus, Palestine; 2 An-Najah BioSciences Unit, Centre for Poisons Control, Chemical and Biological Analyses, An-Najah National University, Nablus, Palestine

**Keywords:** Epilepsy, Consensus, Education, Knowledge, Nurses

## Abstract

**Background:**

Knowledge deficits with regard to epilepsy have been reported among healthcare professionals. This study was conducted to develop consensus-based aims, contents, intended learning outcomes, teaching, and evaluation methods for a course on epilepsy for postgraduate or continuing education in community health nursing programs.

**Methods:**

A mixed method which combined a thorough search of literature, the nominal group technique, the Delphi technique, and survey of students’ agreement was used. The databases MEDLINE/PUBMED, EMBASE, COCHRANE, CInAHL/EBESCO, SCOPUS, Google Scholar, Google Books, and Amazon were searched to identify potential aims, topics/contents, intended learning outcomes, teaching, and evaluation methods. Discussions and deliberations in serial meetings based on the nominal group technique were attended by educators/academicians (*n* = 12), neurologists (*n* = 2), practicing nurses (*n* = 5), pharmacists (n = 2), patients with epilepsy (n = 2), and students in postgraduate and continuing education programs (*n* = 7) to supplement and refine the data collected from the literature. The qualitative data were analyzed using RQDA tool for R. The Delphi technique was used among educators/academicians (*n* = 15), neurologists (*n* = 2), practicing nurses (*n* = 5), pharmacists (n = 2), patients with epilepsy (*n* = 3), and students in postgraduate and continuing education programs (*n* = 8) to achieve formal consensus.

**Results:**

Consensus was achieved on 6 aims, 16 intended learning outcomes, and 27 topics in the course. Of the topics, 13 were relevant to nature of epilepsy and seizures, 2 were relevant to the impact of epilepsy and seizures on different life aspects of patients with epilepsy, 4 were relevant to advocating for the patients and supporting their choices, 5 were relevant to educating patients and their caregivers, and 3 were relevant to assessments and services.

**Conclusion:**

Consensus-based aims, topics/contents, intended learning outcomes, teaching, and evaluation methods of a course on epilepsy for postgraduate or continuing education in community health nursing programs were developed. Consensus-based courses could bridge knowledge gaps and improve educating community health nursing programs on epilepsy. Further studies are needed to determine if such consensus-based courses could promote care of patients with epilepsy.

**Supplementary Information:**

The online version contains supplementary material available at 10.1186/s12909-021-03001-2.

## Background

Epilepsy is a complex neurological condition of the brain that is characterized by abnormal electrical discharges from some neurons in the cortex that often result in recurrent seizures [1]. Epilepsy is the second most prevalent neurological condition after stroke and, today, there are more than sixty-five million people who live with epilepsy around the globe [1–3]. According to the World Health Organization (WHO), about 6.5 million people with epilepsy reside in the Eastern Mediterranean region [4].

It is well-stablished that some patients with epilepsy whose seizures are not controlled might need to receive highly specialized services from epileptologists/neurologists who are the most qualified healthcare professionals to care for patients with epilepsy. However, a sustainable care model based on epileptologists/neurologists is not currently feasible. Additionally, the majority of patients with epilepsy do not have conditions that require continuous access to epileptologists/neurologists [5, 6]. Therefore, many patients with epilepsy often receive care from other healthcare providers like general practitioners, internists, pharmacists, and nurses [5–8].

In modern healthcare delivery systems, multi-healthcare provider approach has been increasingly promoted in caring for patients with disabling health conditions including epilepsy [9, 10]. In all healthcare systems around the globe, nurses are important healthcare professionals who are responsible for the provision of large volumes of services to patients including those with epilepsy [10, 11]. In today’s healthcare systems, nurses provide healthcare services to patients in primary, secondary, tertiary, and within highly specialized domains of healthcare settings [12]. Nurses also became part of multi-disciplinary healthcare teams in centers that provide comprehensive and holistic healthcare to patients with epilepsy [10, 11, 13–15].

To respond to the ever-expanding roles and responsibilities of nurses, nursing education has evolved to provide postgraduate specialty training and continuing education for nurses in almost all fields of care like community health [16], mental health [17], intensive/critical care [18], cardiology [19], diabetes [20], anesthesia [21], oncology [22], and neurology [23]. Within some specialties, subspecialities are also offered. For example, within the field neurology, Parkinson’s disease [24], multiple sclerosis [25], and epilepsy postgraduate specialist nursing programs are also offered [26]. The importance of specialist nurses in caring for patients has been emphasized by many researchers, professional groups, healthcare authorities, and patient advocacy groups [8, 10, 26, 27].

Although many educational/training institutions around the world offer postgraduate specialty education/training for nurses, it is noteworthy mentioning that neurology and epilepsy specialist nursing programs are offered by a small number of educational/training institutions around the world. On the other hand, postgraduate and continuing education programs in community health nursing are widely offered by many educational/training institutions around the globe. These programs are often designed to educate/train nurses to care for, support, and empower patients and their families/caregivers [28]. Therefore, nurses would be required to assume responsibility and play a wide range of roles including educating patients and/or their families and caregivers to self-management, ensuring adherence to taking medications, and providing psychosocial care and support [14, 15]. Nurses also play an important role in shaping perceptions of the patients, families/caregivers, and general public about diseases and patients [10].

On a global level, efforts have been surmounting to benchmark and improve knowledge of healthcare providers with regard to seizures and epilepsy [5, 29, 30]. The International League Against Epilepsy (ILAE), the WHO, and the International Bureau for Epilepsy (IBE) initiated the campaign “Out of the Shadow” [31]. The campaign aimed to improve acceptability of people with epilepsy, improve therapy and control over seizures, prevent recurrence of seizures, and improve support and services provided to patients with epilepsy. In Scotland, Stuart and Muir surveyed general practitioners to determine what the physicians wanted to learn about epilepsy and assess their preferences with regard to the teaching program [32]. In another study, a formal consensus technique was used to develop a core list of important knowledge items that community pharmacists need to know about epilepsy [33]. Recently, the Task Force for Epilepsy Education of the ILAE developed a competency-based curriculum in epileptology [34]. The curriculum covered 7 domains, with 42 competencies, and 124 learning objectives. The curriculum was developed in three levels: entry, proficiency, and advanced proficiency. While et al. developed and validated a scale for learning needs assessment for multiple sclerosis specialist nurses [25]. Similar scales to assess learning needs assessment for neurology and/or epilepsy specialist nurses were not developed before.

Currently, many nursing schools offer postgraduate and continuing education in community health nursing. Many postgraduate and continuing education programs in community health nursing offer elective courses that students/trainees can choose from. A few elective courses on epilepsy are currently offered in postgraduate and continuing education programs in community health nursing. The present study was conducted to develop consensus-based aims, contents, intended learning outcomes, teaching, and evaluation methods for a course on epilepsy for postgraduate or continuing education in community health nursing programs. This study has scientific, academic, and practical relevance that can be extended beyond the geographical location within which it was conducted. The developed course can be used or modified as appropriate in educating/training community health nurses elsewhere and the methodology can be used in developing other courses to be offered in undergraduate and postgraduate education/training.

## Methods

### Design of the study

**Fig. 1:**
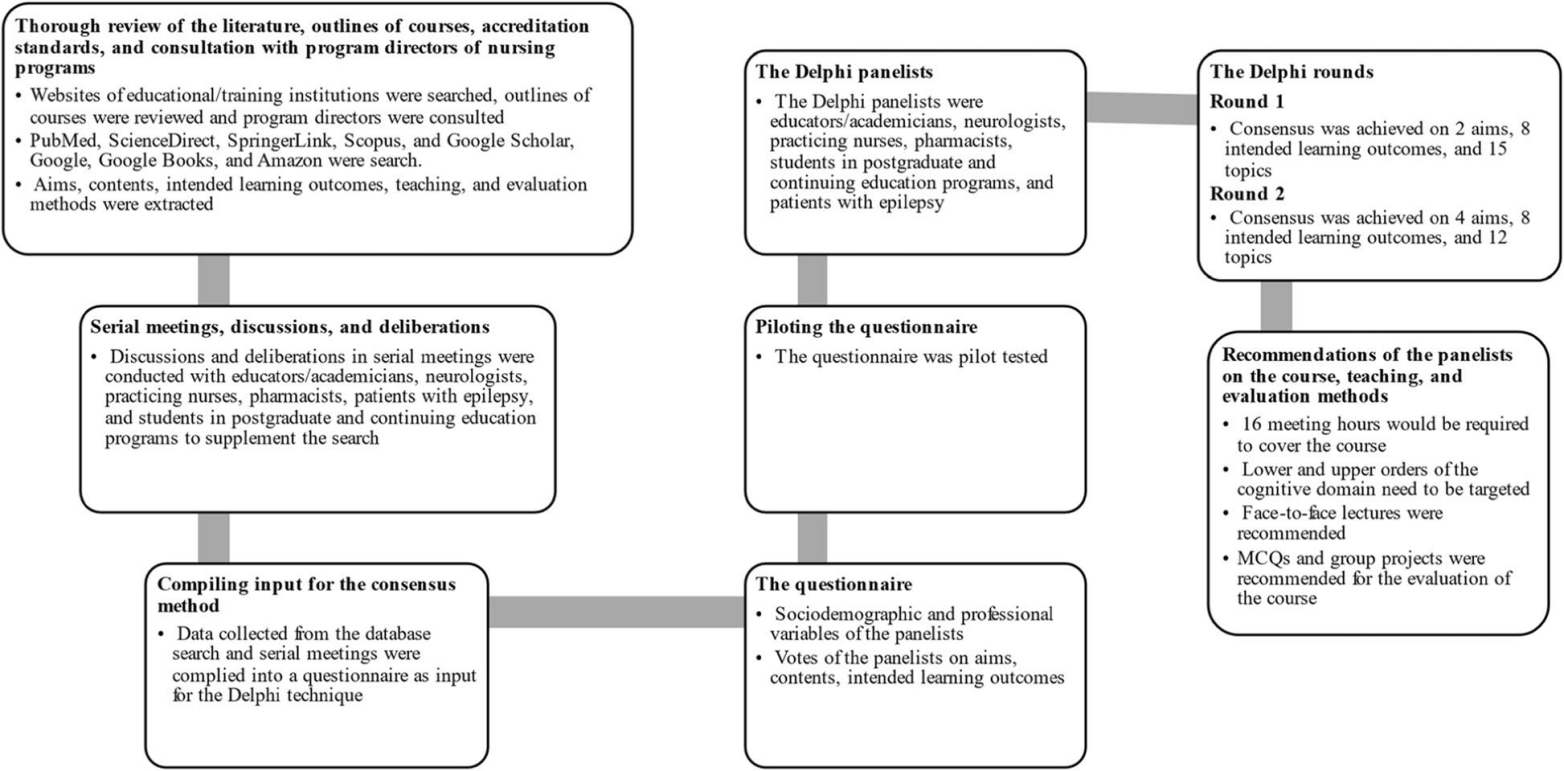
Flow diagram illustrating the different stages of the study

In the present study, a mixed method was used to achieve the study objectives. The nominal group technique was conducted in adherence with the COnsolidated Criteria for REporting Qualitative Research (COREQ) Checklist [35] and the Delphi technique was conducted in adherence to the Conducting and REporting of DElphi Studies (CREDES) guidelines [36]. Adherence to the COREQ Checklist is shown in Supplementary Table S1 and adherence to the CREDES checklist is shown in Supplementary Table S2. In this study, a thorough search of the literature was conducted to collect aims, topics/contents, intended learning outcomes, teaching, and evaluation methods for a course on epilepsy for postgraduate or continuing education in community health nursing programs. Group discussions and deliberations that were based on the nominal group technique were applied in consecutive rounds of meetings with educators/academicians, neurologists, practicing nurses, pharmacists, patients with epilepsy, and students in community health nursing programs to supplement, sort, and tabulate the aims, topics/contents, intended learning outcomes, teaching, and evaluation methods. Formal consensus was sought through two Delphi technique rounds to develop the final consensus-based course. The different stages of the study are shown in Fig. 1.

### Thorough review of the literature, outlines of courses, accreditation standards, and consultation with program directors of nursing programs

The websites of educational/training institutions were searched for outlines of courses offered in community health nursing programs. Outlines of courses were reviewed and program directors of the postgraduate and continuing education programs (*n* = 8) were contacted and consulted with regard to deficiency of courses on epilepsy.

To identify relevant aims, topics/contents, intended learning outcomes, teaching, and evaluation methods for a course on epilepsy, a thorough search and review of literature was conducted. The search was performed through the following databases: MEDLINE through PUBMED, EMBASE, COCHRANE, CInAHL hosted by EBSCO, and SCOPUS. The MeSH terms related to “epilepsy*” and “education” were mainly used. The search strategy was informed by previous search strategies. Documents retrieved from the databases were supplemented with a search for books using Google Scholar, Google Books, and Amazon. Documents retrieved through the search were compiled. Data that were potentially relevant to aims, topics/contents, intended learning outcomes, teaching, and evaluation methods were extracted into a data extraction form.

### Recruitment of the panelists

A purposive sampling technique was followed to invite and recruit panelists to the nominal group and Delphi technique rounds. The sampling technique used in this study was informed by previous studies that use the two techniques [33, 37]. The panelists were identified and contacted through key contacts in the field. As currently there is no consensus on an ideal panel size, the panel size in this study was informed by previous studies [33, 37]. Having prior knowledge of the subject being investigated was a prerequisite for being selected into the panel. For this study, the educators/academicians were invited based on their previous practice in teaching courses relevant to epilepsy. The healthcare professionals were invited based on their involvement in caring for patients with epilepsy. Patients with epilepsy and students in postgraduate and continuing education programs were also included in the panel to ensure representation of these groups in the panel. Before the commencement of the meetings, the design and objectives of the study were explained to the participants and their written informed consents were obtained.

### Serial meetings, discussions, and deliberations

Discussions and deliberations in serial meetings were conducted as part of a nominal group technique. The meetings were moderated by the main investigator (PhD) who was employed by a major teaching university at the time of the study. The main investigator had experience in moderating nominal group meetings and conducting qualitative research. The meetings were attended by educators/academicians (*n* = 12), neurologists (*n* = 2), practicing nurses (*n* = 5), pharmacists (n = 2), patients with epilepsy (n = 2), and students in postgraduate and continuing education programs (*n* = 7). Of the 12 educators/academicians who participated in the nominal group meetings, 8 were program directors who were responsible for oversight of postgraduate/continuing education programs. The nominal group technique was used in this study because it resembles the way discussions and deliberations are conducted to make decisions in curricula development committees and advisory boards [38]. For this study, the checklist developed by Humphrey-Murto et al. was used to ensure rigor of the methodology [39]. The objective of these meetings was to collect additional aims, intended learning outcomes, topics/contents, teaching, and evaluation methods that were not retrieved from the literature search. The meetings (*n* = 4) were recorded and transcribed verbatim.

### Analysis of the qualitative data

A thematic analytical method based on the interpretive description methodology was used to analyze the contents of the transcripts [40]. The method allowed recognizing themes and subthemes. Additionally, the Qualitative Analysis Guide of Leuven was used in the recognition of themes and subthemes [41]. The RQDA tool for R was used in the analysis of the qualitative data [42].

### Compiling input for the consensus method

Aims, topics/contents, intended learning outcomes, teaching, and evaluation methods that were retrieved from the published literature and those provided by the educators/academicians, neurologists, practicing nurses, pharmacists, students, and patients with epilepsy in the nominal meetings were compiled into a questionnaire. The questionnaire collected the sociodemographic and academic/practice characteristics of the participants. Additionally, aims, intended learning outcomes, and contents/topics were listed and the panelists were asked to express their disagreement/agreement on each item using a Likert-scale of 1–9 (1 indicated complete disagreement, 9 indicated complete agreement).

### Pilot testing of the questionnaire

The questionnaire was pilot tested with 2 educators/academicians, 2 practicing nurses, and 3 students. This pilot testing aimed to ensure clarity and comprehensibility of the questionnaire. Some items of the questionnaire were rephrased to improve clarity.

### The consensus method

In this study, the Delphi technique was used to achieve consensus on the aims, intended learning outcomes, and topics/contents of the course. The Delphi technique has evolved as one of the most commonly used formal consensus techniques in healthcare and curriculum development. The panelists were educators/academicians (*n* = 15), neurologists (*n* = 2), practicing nurses (*n* = 5), pharmacists (n = 2), patients with epilepsy (*n* = 3), and students in postgraduate and continuing education programs (*n* = 8). The Delphi panelists were approached and recruited using key contacts in the field using similar inclusion criteria that were used in the recruitment of the panelists for the nominal group.

### Delphi round 1

In the Delphi round 1, the panelists were asked to respond to the questionnaire by providing their sociodemographic and academic/practice details and expressing their disagreement/agreement on each item using the Likert-scale of 1–9 (1 = complete disagreement, 9 = complete agreement).

### Analysis of ratings and consensus definition

The ratings of the panelists were entered into a Microsoft Excel Spreadsheet (Microsoft Inc.) and descriptive statistics like lower quartile (Q1), median (Q2), upper quartile (Q3), and the interquartile range (IQR) were used to analyze the data. The definition of consensus that was used in this study was informed by previous studies in which the Delphi technique was used to achieve consensus on concepts in healthcare. Consensus in this study was as follows: 1) the median rating was within the range 7–9 and the interquartile range (IQR) was ≤2, consensus was said to have been achieved and the item was included, 2) the median rating was within the range 1–3 and the IQR was ≤2, consensus was said to have been achieved and the item was excluded. On the other hand, when the median rating was within the range 4–6 or the IQR was > 2, the item was considered equivocal. As in previous studies, it was decided a priori that equivocal items would be carried forward into a subsequent Delphi round (round 2).

### Delphi round 2

The items that were considered as equivocal in the Delphi round 1 were carried forward into the Delphi round 2. The panelists were provided with a revised questionnaire that contained the equivocal items in addition to a remind of their own rating on each item, the median, and the IQR of ratings of other panelists. The panelists were asked if they wished to reconsider their ratings after considering the ratings of the other panelists. Ratings of the Delphi round 2 was analyzed using the same definition in the Delphi round 1.

### Proposed course overview, teaching, and evaluation methods

Based on the votes of the panelists, the principal investigator proposed the course overview, teaching, and evaluation methods. The proposed plan was sent to the panelists for review and comments. The panelists were asked to submit their detailed review and comments on the proposed the course overview, teaching, and evaluation methods. The panelists were also encouraged to include their suggestions, views, and opinions. Comments and suggestions of the panelists were analyzed qualitatively as described in the section Analysis of the qualitative data.

### Ethics approval and consent to participate

This study was conducted in adherence with the principles of the Declaration of Helsinki and the ethical principles followed at An-Najah National University. The current study received ethical approval from the Institutional Review Board (IRB) of An-Najah National University. All participants provided written informed consent.

## Results

### Sociodemographic and academic/practice variables of the participants

Of the nominal group participants, 18 (60.0%) were male, 16 (53.3%) were 40 years and older, 9 (30.0%) were practicing healthcare professionals (neurologists, nurses, and pharmacists). Of the educators/academicians and practicing healthcare professionals, 16 (76.2%) had 10 or more years of practicing experience. Of the Delphi panelists, 20 (57.1%) were male, 19 (54.3%) were 40 years and older, 15 (42.9%) were educators/academicians, 2 (5.7%) were neurologists, 5 (14.3%) were practicing nurses, 2 (5.7%) were pharmacists, and 3 (8.6%) were patients with epilepsy. The detailed sociodemographic and academic/practice variables of the panelists are shown in Table 1.

**Table 1:** Sociodemographic and academic/practice variables of the panelists

Variable	Nominal group		Delphi panel	
	n	%	n	%
**Gender**				
Male	18	60.0	20	57.1
Female	12	40.0	15	42.9
**Age (years)**				
< 40	14	46.7	16	45.7
≥ 40	16	53.3	19	54.3
**Specialty/status**				
Educator/academician	12	40.0	15	42.9
Neurologist	2	6.7	2	5.7
Nurse	5	16.7	5	14.3
Pharmacist	2	6.7	2	5.7
Postgraduate student	7	23.3	8	22.9
Patient with epilepsy	2	6.7	3	8.6
**Academic degree**				
BSc	6	20.0	8	22.9
MSc^a^	10	33.3	10	28.6
MD	2	6.7	2	5.7
PhD	12	40.0	15	42.9
**Employer**				
Education/training institution^a^	22	73.3	25	71.4
Hospital	6	20.0	6	17.1
Private sector	2	6.7	4	11.4
**Number of years in practice**^b^				
5–9	5	23.8	7	29.2
≥ 10	16	76.2	17	70.8

*BSc* Bachelor of Science, *MSc* Master of Science, *MD* Doctor of Medicine, *PhD* Doctor of Philosophy, ^a^Because students were in their MSc program in an academic institution, they were added to this category, ^b^Calculations were based on the number of educators/academicians, neurologists, nurses, and pharmacists who participated in the nominal group (*n* = 21) and the Delphi panel (*n* = 24)

### Consensus-based aims

In the first Delphi round, consensus was achieved on 2 aims of the course. In the second Delphi round, consensus was achieved on further 4 aims. The consensus-based aims of the course are shown in Table 2.

**Table 2:** Consensus-based aims of the course

#	Aim	Round 1	Round 2
		Median [Q1, Q3]	Median [Q1, Q3]
1	Providing community health nurses with a thorough knowledge and understanding of the differing etiologies of epilepsy and seizures	7 [6, 9]	8 [7, 9]
2	Providing community health nurses with a thorough understanding of how different therapeutic options can control seizures	8 [7, 8]	na
3	Acquainting community health nurses how to advocate and educate patients with epilepsy and/or their caregivers/families with regard to diagnosis, impact of epilepsy on their lives, how to self-manage, and/or access coordinated care	7 [7, 8]	na
4	Acquainting community health nurses how to assist patients with epilepsy and/or their caregivers to function optimally within constraints of their illness through timely access to advice, diagnostic examination, and medications/therapeutic options	7 [5, 8]	7 [7, 8]
5	Enabling community health nurses to educate colleagues, trainees, communities, and lead quality improvement projects and research	7 [5, 8]	7 [7, 8]

*Q1* Lower quartile, *Q3* Upper quartile, *na* Not applicable

### Consensus-based intended learning outcomes

In the first Delphi round, consensus was achieved on 8 intended learning outcomes of the course. In the second Delphi round, consensus was achieved on further 8 intended learning outcomes. The consensus-based intended learning outcomes of the course are shown in Table 3.

**Table 3:** Consensus-based intended learning outcomes of the course

#	Outcome	Round 1	Round 1
		Median [Q1, Q3]	Median [Q1, Q3]
1	Describe the pathophysiological basis of epilepsy and seizures	5 [4, 7]	7 [6, 8]
2	Describe the different types of epilepsies and seizures associated with each type	6 [5, 7]	7 [6, 8]
3	Explain the mechanism of action of the different therapeutic options available to control seizures	8 [7, 8]	na
4	Evaluate alternative therapeutic options taking into consideration their potential efficacies and adverse effects	8 [7, 8]	na
5	Reflect on the importance of adherence to taking antiepileptic medications	8 [7, 9]	na
6	Recognize the different adverse effects of antiepileptic drugs	8 [7, 9]	na
7	Discuss how to optimize therapy and minimize the incidence of adverse effects of antiepileptic drugs	8 [7, 8]	na
8	Recognize drug-drug, drug-food, and drug-herbal interactions relevant to antiepileptic drugs	7 [6, 8]	na
9	Reflect on the impact of epilepsy and seizures on the various aspects of social life of the patients	6 [4, 7]	7 [5, 7]
10	Design and evaluate personalized care plans for individual patients with epilepsy	6 [4, 8]	7 [5, 7]
11	Assess eligibility of patients with epilepsy for social and economic support	5 [4, 8]	7 [5, 7]
12	Design and evaluate personalized educational materials for individual patients with epilepsy	6 [4, 7]	7 [5, 7]
13	Discuss the importance of keeping a record of seizures to document their frequency, time of occurrence, and potential triggers for each individual patient	5 [4, 8]	7 [5, 7]
14	Demonstrate general safety procedures and first aid for patients while experiencing seizures	7 [6, 8]	na
15	Reflect on the importance of managing comorbidities like anxiety and depression on the mental health of patients with epilepsy	6 [4, 7]	7 [5, 7]
16	Demonstrate assessment methods for injuries and trauma that could be associated with seizures	8 [7, 8]	na

*Q1* Lower quartile, *Q3* Upper quartile, *na* Not applicable

### Consensus-based course contents/topics

In the first Delphi round, consensus was achieved on 15 topics. In the second Delphi round, consensus was achieved on further 12 topics. In both rounds, consensus was achieved on 13 items relevant to nature of epilepsy and seizures, 2 items relevant to the impact of epilepsy and seizures on different life aspects of patients with epilepsy, 4 items relevant to advocating for the patients and supporting their choices, 5 items relevant to educating patients and their caregivers, and 3 items relevant to assessments and services. Details of the consensus-based topics are shown in Table 4.

**Table 4:** Consensus-based course contents/topics

#	Content/topic	Round 1	Round 1
		Median [Q1, Q3]	Median [Q1, Q3]
**Contents relevant to nature of epilepsy and seizures**			
1	Incidence and prevalence rates at global level and in particular communities.	6 [4, 8]	7 [6, 8]
2	Pathophysiology of epilepsy and seizures.	8 [7, 9]	na
3	Epilepsy as a spectrum: types of epilepsies and their associated seizures.	8 [6, 8]	na
**Contents relevant to management/treatment of seizures**			
4	Therapeutic options available for patients with epilepsy.	8 [7, 8]	na
5	The mechanism of action of antiepileptic drugs.	7 [6, 7]	na
6	Selection of antiepileptic drugs and therapeutic goals.	8 [7, 8]	na
7	Administering antiepileptic therapy.	7 [6, 7]	na
8	Assessing effectiveness of antiepileptic therapy.	6 [4, 8]	7 [6, 8]
9	Ensuring adherence of patients to taking their antiepileptic therapy.	7 [6, 7]	na
10	Adverse effects of antiepileptic therapy.	8 [7, 8]	na
11	Screening for and minimizing adverse effects of antiepileptic therapy.	8 [7, 8]	na
12	Drug interactions of antiepileptic therapy.	7 [6, 7]	na
13	Screening for and minimizing drug-drug, drug-food, and drug-herbal interactions.	7 [6, 7]	na
**Contents relevant to the impact of epilepsy and seizures on different life aspects of patients with epilepsy**			
14	Impact of epilepsy and seizures on the social life of patients including relationships, marriage, having children, and driving.	6 [4, 8]	8 [7, 9]
15	Impact of epilepsy and seizures on the economic life of patients including employment.	6 [5, 8]	7 [6, 8]
**Contents relevant to advocating for the patients and supporting their choices**			
16	Supporting decisions of patients on a personalized care plan though communication, trust, and empowered decisions.	6 [4, 8]	7 [6, 8]
17	Assessing eligibility of patients for social and economic support.	6 [4, 8]	7 [6, 8]
18	Supporting choices of patients in compliance with their personal, cultural, life style, and faith values.	6 [4, 8]	7 [6, 8]
19	Supporting patients in coping with their disease and adverse effects of their treatment.	7 [6, 7]	na
**Contents relevant to educating patients and their caregivers**			
20	Educating patients on their disease, seizures, and treatment options.	6 [4, 8]	7 [6, 7]
21	Educating patients how to keep a record of seizures to document their frequency, time of occurrence, and potential triggers.	6 [4, 8]	7 [6, 8]
22	Educating patients on triggers of seizures and how to reduce the number and frequency of seizures.	7 [6, 7]	na
23	Educating the general caregivers and the general public on the nature of epilepsy and seizures and improving their attitudes toward people with epilepsy.	6 [4, 8]	8 [7, 9]
24	Educating caregivers on general safety procedures and first aid for patients while experiencing seizures.	8 [7, 8]	na
**Contents relevant to assessments and services**			
25	Periodic assessments of care plans.	8 [7, 8]	na
26	Assessing the mental health of patients for presence of manageable issues like anxiety and depression.	6 [5, 8]	7 [6, 8]
27	Assessing the physical health of patients for presence of manageable issues injuries and trauma.	6 [4, 8]	7 [6, 8]

*Q1* Lower quartile, Q3 Upper quartile, IQR: *na* Not applicable

### Thematic analysis

The thematic analysis of the contents of the meetings resulted in a number of themes and subthemes relevant to the number of meeting hours, methods of teaching, and evaluation of the course. The themes, subthemes, and sample quotations are shown in Table 5.

**Table 5:** Themes, subthemes, and sample quotations

Theme	Subtheme	Sample quotation
**Number of meeting hours**		“… allocating sufficient time to cover the teaching materials is crucial for the success of the course. I think instructors will need about 32 meeting hours to cover the course materials.” An educator/academician with 15 years of professional and teaching experience
**Design of the course**	**Targeting lower and upper orders of the cognitive domain**	“… the course should be designed to gradually target lower and upper orders of the cognitive domain. Bloom’s verbs should be appropriately used to ensure engaging students.” An educator/academician with 11 years of academic and research experience
	**High quality course**	“… yes, I agree! The course should be designed to prepare competent nurses who would need to care for the increasing number of epilepsy patients” A nurse with 14 years of practicing experience
**Teaching methods**	**Approach**	“In my opinion, face-to-face meeting is superior to all other approaches to teaching. Face-to-face lectures are important for the success of this course” An educator/academician with 9 years of professional, teaching, and research experience
	**Using visual/auditory aids**	“Lecturers have plenty of options to choose from. I would use PowerPoint presentations. I know some colleagues who even use animations.” An educator/academician with 13 years of professional, teaching, and research experience
		“… in case we would not take the students to the hospital or clinic to see real cases, we have a simulations lab. Videos can also be helpful … “An educator/academician with 10 years of professional, teaching, and research experience
**Evaluation methods**	**MCQs**	“MCQs would work well for such a course. MCQs are widely used in licensure examinations.” A nurse with 14 years of teaching and practicing experience
	**Group project**	“I think a group project to be evaluated by the instructor would be a good end of this course” A practicing nurse with 9 years of experience

### Number of meetings and methods of evaluation

During the discussions and deliberations, the panelists suggested that the course contents could be covered in 32 h of dedicated meetings. A representative quotation is shown in Table 5.

### Using Bloom’s taxonomy

**Fig. 2:**
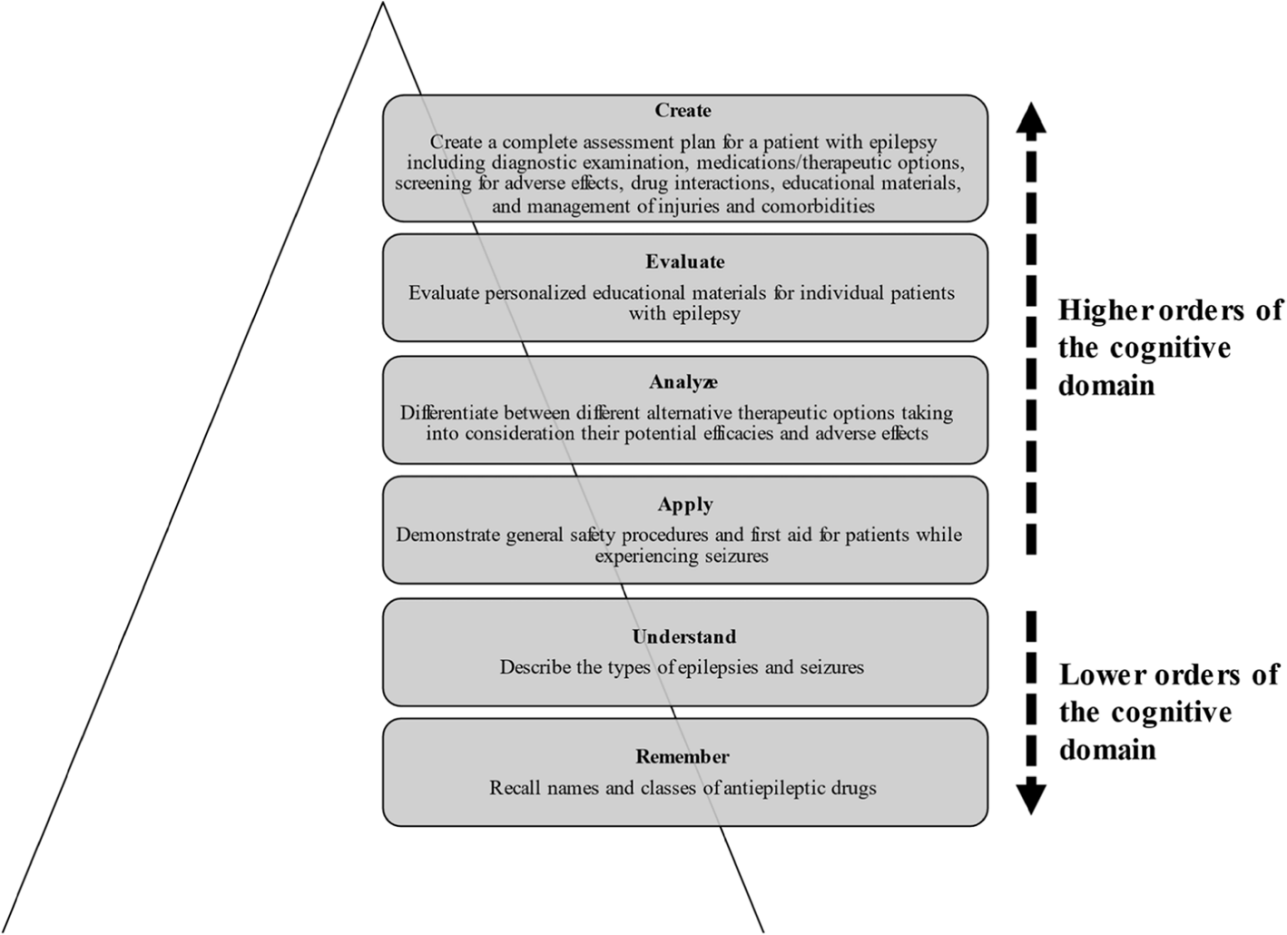
Targeting lower and upper orders of the cognitive domain through Bloom’s verbs

The panelists who participated in the discussions and deliberations suggested that students should be engaged targeting lower and upper orders of the cognitive domain on Bloom’s taxonomy. Some representative quotations are shown in Table 5. Bloom’s verbs like describe (knowledge) and explain (understand) were used in the targeting of the lower orders of the cognitive domain. On the other hand, Bloom’s verbs like reflect (evaluate) were used in the targeting of the upper orders of the cognitive domain. The use of Bloom’s verbs to target lower and upper orders of the cognitive domain is shown in Fig. 2.

### Teaching methods

In this study, the panelists who participated in the discussions and deliberations suggested that the course could be taught through face-to-face lectures. Some representative quotations are shown in Table 5. During the lectures, instructors might be encouraged to present the study materials through Preezi or PowerPoint presentations.

**Fig. 3:**
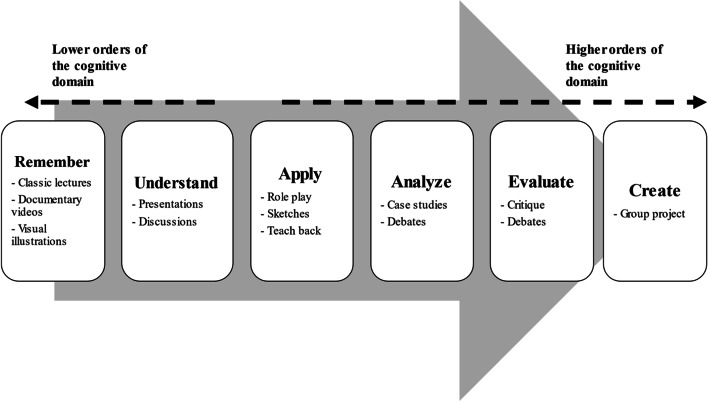
Targeting upper and lower orders of the cognitive domain in teaching and evaluating students through Bloom’s taxonomy

Instructors should also provide reading materials that students need to prepare in advance and engage in group discussions. Videos, audios, and case studies might also be presented and students might be encouraged to engage in discussions. Students should also be encouraged to write their reflections on the cases. Group discussions, group projects, discussions, debates, and written reflections might aid applying knowledge and target upper orders of the cognitive domain. Figure 3 shows how Bloom’s taxonomy can be applied in teaching the course and evaluation of the students.

### Evaluation methods

The panelists who participated in the discussions and deliberations suggested that multiple-choice questions (MCQs) can be used to assess lower and upper orders of the cognitive domain, as appropriate. Additionally, Group discussions, group projects, discussions, debates, and written reflections might be used to evaluate the upper orders of the cognitive domain. Figure 3 shows how the lower and upper orders of the cognitive domain might be evaluated.

## Discussion

In this study, consensus-based aims, topics/contents, intended learning outcomes, teaching, and evaluation methods for a course on epilepsy for postgraduate or continuing education in community health nursing programs were developed for the first time using a mixed method. The 27 consensus-based contents covered materials relevant to the nature of epilepsy, impact of epilepsy and seizures on the lives of patients, advocating for patients and supporting their choices, educating patients and their families/caregivers, and assessments and services. Currently, little guidance is available on what topics/contents should be covered in a course on epilepsy for postgraduate or continuing education in community health nursing programs. Findings of this study might provide a core list of consensus-based topics/contents that could be helpful in guiding educators/trainers in designing courses/curricula for postgraduate or continuing education in community health nursing programs.

To achieve the objectives of this study, a mixed method was used. Aims, topics/contents, intended learning outcomes, teaching, and evaluation methods were retrieved from published documents following a search of databases and search engines. In this study, a thorough search and review of the literature was used instead of a systematic review of the literature taking into consideration the nature of the study, objectives, scope, research questions, problem/population, intervention, comparison, outcome, and study design (PICOS), in addition to the nature of the documents to be retrieved [43]. Additionally, the websites of educational/training institutions offering postgraduate and/or continuing education programs were searched and outlines of courses were reviewed. Directors of postgraduate and continuing education in community health nursing were consulted. Aims, topics/contents, intended learning outcomes, teaching, and evaluation methods retrieved from the literature were supplemented by more items provided by the participants in the nominal group meetings. Educators, neurologists, practicing nurses, pharmacists, and patients with epilepsy who took part in the discussions and deliberations of the nominal group serial meetings were rich in knowledge and information about epilepsy. Discussions and deliberations of the nominal group helped refining the compiling the list of potential aims, topics/contents, intended learning outcomes and added depth and width to those collected from the literature.

Students in postgraduate and continuing education in community health nursing were also included in the deliberations, discussions, and the Delphi panel. Views and opinions of students/learners are increasingly considered when developing and designing courses/curricula. To design a course on epilepsy for general practitioners in Scotland, Stuart and Muir collected views of the general practitioners to determine what they wanted to learn and to collect their preferences with regard to the teaching method [32]. Similarly, modern teaching approaches aim to engage students in the learning process [44]. Currently, gold-standard in designing courses for undergraduate nursing students do not exist. In the absence of gold standards, engaging experts who are rich in information like educators, neurologists, practicing nurses, and patients with epilepsy could provide suitable alternatives in designing courses/curricula on epilepsy for postgraduate or continuing education in community health nursing programs [33, 37]. Taking into consideration the views and opinions of the students to achieve formal consensus on topics/contents of the course could be appealing to the learners as it has been argued that students are more likely to engage in a course they agree with its contents than a course they did not participate in its design and selection of its contents.

Considerably, the number of participants who took part in this study was larger than those used in previous consensus developing approaches in healthcare [45, 46]. Currently, there is no consensus on a suitable panel size in formal consensus methods. Previous studies have used panels in the sizes of less than 70 panelists [33, 37, 45, 46]. The panelists who participated in this study were diversified in terms of their sociodemographic and practice variables. Such diversity might add strength and validity to the findings reported in this study [36]. This might also increase the likelihood of using the consensus-based topics/contents in courses/curricula on epilepsy for postgraduate or continuing education in community health nursing programs.

In general, the consensus-based topics/contents in this study were consistent with the ever unfolding roles and responsibilities of nurses in caring for patients with epilepsy as seen by professional nursing associations and international organizations like the ILAE, IBE, and the WHO [13, 34, 47]. The depth and width of the topics/contents on which consensus was achieved in this study might guide educators and trainers in designing courses/curricula on epilepsy for postgraduate or continuing education in community health nursing programs.

### Strengths and limitations

The present study has a number of strengths and limitations that need to be considered while interpreting the findings. First, consensus-based aims, topics/contents, intended learning outcomes, teaching, and evaluation methods for a course on epilepsy was developed for the first time. Second, a mixed method that combined a thorough search of the literature, nominal group technique, the Delphi technique, and survey was used. Third, the panelists were diversified in terms of specialty and sociodemographic and practice characteristics. In this study, educators, neurologists, practicing nurses, pharmacists, students in postgraduate community health nursing programs and patients with epilepsy participated in the development of the course.

On the other hand, this study has a number of limitations. First, a systematic literature review was not followed to identify aims, topics/contents, intended learning outcomes, teaching, and evaluation methods that potentially could be included in the consensus-based course. Compared with thorough reviews of the literature, systematic reviews are robust and provide reproducible results. Second, although different methods were combined to achieve the objectives of the study, qualitative methods are inherently limited. Findings are opinions and reflect thoughts of the experts who provided them. Third, consensus was valid within the definitions used. However, using different definitions of consensus could have resulted in different findings.

## Conclusion

Consensus-based aims, topics/contents, intended learning outcomes, teaching, and evaluation methods of a course on epilepsy for postgraduate or continuing education in community health nursing programs were developed. Consensus-based course contents are important tools in guiding the development and design of courses/curricula aiming to engaging learners. Consensus-based courses could bridge knowledge gaps and improve educating community health nursing programs on epilepsy. Further studies are needed to determine if such consensus-based courses could promote care of patients with epilepsy.

## Supplementary Information

**Additional file 1: Supplementary Table S1:** Adherence to COnsolidated criteria for REporting Qualitative research (COREQ) Checklist [35]. **Supplementary Table S2:** Adherence to Conducting and REporting of DElphi Studies (CREDES) guidelines [36].

## Data Availability

The data associated with this study are included within the manuscript. The datasets used in the analysis in this study are available from the corresponding author on reasonable request.
