# Supplementary material for: Using a mixed method to develop consensus-based aims, contents, intended learning outcomes, teaching, and evaluation methods for a course on epilepsy for postgraduate or continuing education in community health nursing programs
Source: BMC Med Educ. 2021 Nov 12;21:572. doi: 10.1186/s12909-021-03001-2 (PMC8588674; doi:10.1186/s12909-021-03001-2)
Supplement: Supplementary file 1 — Additional file 1: Supplementary Table S1: Adherence to COnsolidated criteria for REporting Qualitative research (COREQ) Checklist [35]. Supplementary Table S2: Adherence to Conducting and REporting of DElphi Studies (CREDES) guidelines [36]. [file 12909_2021_3001_MOESM1_ESM.docx]

**Using a mixed method to develop consensus-based aims, contents, intended learning outcomes, teaching, and evaluation methods for a course on epilepsy for postgraduate or continuing education in community health nursing programs**

Ramzi Shawahna^1,2*^

^1^Department of Physiology, Pharmacology and Toxicology, Faculty of Medicine and Health Sciences, An-Najah National University, Nablus, Palestine

^2^An-Najah BioSciences Unit, Centre for Poisons Control, Chemical and Biological Analyses, An-Najah National University, Nablus, Palestine

**^*^Correspondence:**

Ramzi Shawahna, PhD, Department of Physiology, Pharmacology and Toxicology, Faculty of Medicine & Health Sciences, New Campus, Building: 19, Office: 1340, An-Najah National University, P.O. Box 7, Nablus, Palestine

Phone: + (970) 923 45113 ext 2772

Phone: + (970) 92349739

Email: [ramzi_shawahna@hotmail.com](mailto:ramzi_shawahna@hotmail.com)

**Supplementary Table S1:** Adherence to COnsolidated criteria for REporting Qualitative research (COREQ) Checklist [[1](#_ENREF_1)]

| **#** | **Topic** | **Guide Questions/Description** | **Section in the manuscript** |
| --- | --- | --- | --- |
|  | **Domain 1: Research team and reﬂexivity** |  |  |
|  | *Personal characteristics* |  |  |
| 1 | Interviewer/facilitator | Which author/s conducted the interview or focus group? | Provided in the Methods section, under sub-section: Serial meetings, discussions, and deliberations |
| 2 | Credentials | What were the researcher’s credentials? E.g. PhD, MD | Provided in the Methods section, under sub-section: Serial meetings, discussions, and deliberations |
| 3 | Occupation | What was their occupation at the time of the study? | Provided in the Methods section, under sub-section: Serial meetings, discussions, and deliberations |
| 4 | Gender | Was the researcher male or female? | Provided in the Methods section, under sub-section: Serial meetings, discussions, and deliberations |
| 5 | Experience and training | What experience or training did the researcher have? | Provided in the Methods section, under sub-section: Serial meetings, discussions, and deliberations |
|  | *Relationship with participants* |  |  |
| 6 | Relationship established | Was a relationship established prior to study commencement? | Provided in the Methods section, under sub-section: Recruitment of the panelists |
| 7 | Participant knowledge of the interviewer | What did the participants know about the researcher? e.g. personal goals, reasons for doing the research | Provided in the Methods section, under sub-section: Recruitment of the panelists |
| 8 | Interviewer characteristics | What characteristics were reported about the inter viewer/facilitator? e.g. Bias, assumptions, reasons and interests in the research topic | Provided in the Methods section, under sub-section: Recruitment of the panelists |
|  | **Domain 2: Study design** |  |  |
|  | *Theoretical framework* |  |  |
| 9 | Methodological orientation and Theory | What methodological orientation was stated to underpin the study? e.g. grounded theory, discourse analysis, ethnography, phenomenology, content analysis | Provided in the Methods section, under sub-section: Analysis of the qualitative data |
|  | *Participant selection* |  |  |
| 10 | Sampling | How were participants selected? e.g. purposive, convenience, consecutive, snowball | Provided in the Methods section, under sub-section: Recruitment of the panelists |
| 11 | Method of approach | How were participants approached? e.g. face-to-face, telephone, mail, email | Provided in the Methods section, under sub-section: Recruitment of the panelists |
| 12 | Sample size | How many participants were in the study? | Provided in the Methods section, under sub-section: Recruitment of the panelists |
| 13 | Non-participation | How many people refused to participate or dropped out? Reasons? | N/A |
|  | *Setting* |  |  |
| 14 | Setting of data collection | Where was the data collected? e.g. home, clinic, workplace | Provided in the Methods section, under sub-section: Serial meetings, discussions, and deliberations |
| 15 | Presence of non-participants | Was anyone else present besides the participants and researchers? | Provided in the Methods section, under sub-section: Serial meetings, discussions, and deliberations |
| 16 | Description of sample | What are the important characteristics of the sample? e.g. demographic data, date | Provided in the Results section, under sub-section: Sociodemographic and academic/practice variables of the participants |
|  | *Data collection* | | |
| 17 | Interview guide | Were questions, prompts, guides provided by the authors? Was it pilot tested? | Provided in the Methods section, under sub-section: Pilot testing of the questionnaire |
| 18 | Repeat interviews | Were repeat interviews carried out? If yes, how many? | Provided in the Methods section, under sub-section: Serial meetings, discussions, and deliberations |
| 19 | Audio/visual recording | Did the research use audio or visual recording to collect the data? | Provided in the Methods section, under sub-section: Serial meetings, discussions, and deliberations |
| 20 | Field notes | Were ﬁeld notes made during and/or after the interview or focus group? | Provided in the Methods section, under sub-section: Serial meetings, discussions, and deliberations |
| 21 | Duration | What was the duration of the inter views or focus group? | Provided in the Methods section, under sub-section: Serial meetings, discussions, and deliberations |
| 22 | Data saturation | Was data saturation discussed? | Provided in the Methods section, under sub-section: Serial meetings, discussions, and deliberations |
| 23 | Transcripts returned | Were transcripts returned to participants for comment and/or correction? | Provided in the Methods section, under sub-section: Serial meetings, discussions, and deliberations |
|  | **Domain 3: analysis and ﬁndings** |  |  |
|  | *Data analysis* | | |
| 24 | Number of data coders | How many data coders coded the data? | Provided in the Methods section, under sub-section: Analysis of the qualitative data |
| 25 | Description of the coding tree | Did authors provide a description of the coding tree? | N/A |
| 26 | Derivation of themes | Were themes identiﬁed in advance or derived from the data? | Provided in the Methods section, under sub-section: Analysis of the qualitative data |
| 27 | Software | What software, if applicable, was used to manage the data? | Provided in the Methods section, under sub-section: Analysis of the qualitative data |
| 28 | Participant checking | Did participants provide feedback on the ﬁndings? | Provided in the Methods section, under sub-section: Serial meetings, discussions, and deliberations |
|  | *Reporting* |  |  |
| 29 | Quotations presented | Were participant quotations presented to illustrate the themes/ﬁndings? Was each quotation identiﬁed? e.g. participant number | Provided in the results section and in Table 5 |
| 30 | Data and ﬁndings consistent | Was there consistency between the data presented and the ﬁndings? | N/A |
| 31 | Clarity of major themes | Were major themes clearly presented in the ﬁndings? | Provided in the results section and in Table 5 |
| 32 | Clarity of minor themes | Is there a description of diverse cases or discussion of minor themes? | Provided in the results section and in Table 5 |

**Supplementary Table S2:**

Adherence to Conducting and REporting of DElphi Studies (CREDES) guidelines [[2](#_ENREF_2)]

| **#** | **Category** | **Section/paragraph in the manuscript** |
| --- | --- | --- |
|  | **Rationale for the choice of the Delphi technique** |  |
| 1 | Justification/rationale for Delphi | Provided in the background and in the Methods Section, under subsection: The consensus method |
| 2 | Purpose well defined | Provided in the background, in the last paragraph |
|  | **Planning and design** |  |
| 1 | Planning and process | Provided in the Methods, subsection: Design of the study |
| 2 | Selection of experts clearly justified | Provided in the Methods, subsection: Recruitment of the panelists |
|  | **Study conduct** |  |
| 1 | Clear description of methods | Methods section. |
| 2 | Flow chart | Figure 1 |
| 3 | Informational input | Methods, Subsection: Compiling input for the consensus method |
| 4 | Clear definition of consensus | Methods, Subsection: Analysis of the votes and definition of consensus |
| 5 | Prevention of bias | Methods, The second Delphi round |
| 6 | Pilot test of instruments | Methods, Subsection: Pilot testing of the questionnaire |
| 7 | Interpretation and processing of results | Methods, Analysis of the votes and definition of consensus |
| 8 | Validity | Methods, Analysis of the votes and definition of consensus |
|  | **Reporting** |  |
| 1 | Transparent reporting of results | Results section. |
| 2 | Data analysis clearly justified and reported | Results, Tables and Figures |
| 3 | Expert panel (Member of organization, recognized authority, relevant clinical academic expertise, profession/stakeholder) | Results, Table 1. |
| 4 | Information of rounds | Results, Consensus-based aims, contents, intended learning outcomes, |
| 5 | Discussion of limitations | Discussion, Strengths and limitations |
| 6 | Adequacy of conclusions | Conclusion |

**References**

1. Tong A, Sainsbury P, Craig J: **Consolidated criteria for reporting qualitative research (COREQ): a 32-item checklist for interviews and focus groups**. *International journal for quality in health care : journal of the International Society for Quality in Health Care* 2007, **19**(6):349-357.

2. Junger S, Payne SA, Brine J, Radbruch L, Brearley SG: **Guidance on Conducting and REporting DElphi Studies (CREDES) in palliative care: Recommendations based on a methodological systematic review**. *Palliative medicine* 2017, **31**(8):684-706.
